# The Treatment of Tuberculous Fistulæ by Potassium Permanganate

**Published:** 1910-07-30

**Authors:** James Scobie

**Affiliations:** Mountstuart, Dollar, N.B.


*To the Editor of* The Hospital.

### THE TREATMENT OF TUBERCULOUS FISTULÆ BY POTASSIUM PERMANGANATE.

*To the Editor of* The Hospital.

Dear Sir,—I thank you for the distinction you have done me in referring to my article in the *Prescriber* on the treatment of tuberculous fistulæ by potassium permanganate. Will you allow me, however, to point out an error in the abstraction? You state that when the residue of the ischio-rectal abscess was opened it discharged "thick" pus. As stated in my article, "thin" pus issued. According to the French writers, and to my somewhat limited experience, the lesions most likely to be benefited by the introduction of pot. permanganate are those of feeble intensity and discharging thin, watery pus. Indeed, I think it established that thick pus is a contra-indication to its use. Possibly, however, you may not be in agreement with this. But in view of my opinion, I thought it better to bring the error before your notice.—I am, Sir, yours faithfully,

Mountstuart, Dollar, N.B., James Scobie, M.B.

July 23, 1910.

